# Polymorphisms in *GEMIN4* and *AGO1* Genes Are Associated with the Risk of Lung Cancer: A Case-Control Study in Chinese Female Non-Smokers

**DOI:** 10.3390/ijerph13100939

**Published:** 2016-09-23

**Authors:** Xue Fang, Zhihua Yin, Xuelian Li, Lingzi Xia, Baosen Zhou

**Affiliations:** 1 Department of Epidemiology, School of Public Health, China Medical University, Shenyang 110122, China; fangxue222@hotmail.com (X.F.); zhyin@mail.cmu.edu.cn (Z.Y.); xlli@mail.cmu.edu.cn (X.L.); xialingzi1013@hotmail.com (L.X.); 2 Key Laboratory of Cancer Etiology and Intervention, University of Liaoning Province, Shenyang 110122, China

**Keywords:** *GEMIN4*, *AGO1*, single nucleotide polymorphism, lung cancer, susceptibility, cooking oil fumes, passive smoking

## Abstract

MicroRNA biosynthesis genes can affect the regulatory effect of global microRNAs to target mRNA and hence influence the genesis and development of human cancer. Here, we selected five single nucleotide polymorphisms (SNPs) (rs7813, rs2740349, rs2291778, rs910924, rs595961) in two key microRNA biosynthesis genes (*GEMIN4* and *AGO1*) and systematically evaluated the association between these SNPs, the gene-environment interaction and lung cancer risk. To control the impact of cigarette smoking on lung cancer, we recruited Chinese female non-smokers for the study. The total number of lung cancer cases and cancer-free controls were 473 and 395 in the case-control study. Four SNPs showed statistically significant associations with lung cancer risk. After Bonferroni correction, rs7813 and rs595961 were evidently still associated with lung cancer risk. In the stratified analysis, our results revealed that all five SNPs were associated with the risk of lung adenocarcinoma; after Bonferroni correction, significant association was maintained for rs7813, rs910924 and rs595961. Haplotype analysis showed *GEMIN4* haplotype C-A-G-T was a protective haplotype for lung cancer. In the combined unfavorable genotype analysis, with the increasing number of unfavorable genotypes, a progressively increased gene-dose effect was observed in lung adenocarcinoma. We also found that individuals exposed to cooking oil fumes showed a relatively high risk of lung cancer, but no interactions were found between cooking oil fume exposure or passive smoking exposure with these SNPs, either on an additive scale or a multiplicative scale. Overall, this is the first study showing that rs7813 and rs595961 could be meaningful as genetic markers for lung cancer risk.

## 1. Introduction

Lung cancer is one of the most common malignant tumors affecting millions of people around the world. In 2012, about 1.8 million lung cancer patients were newly diagnosed, accounting for about 13% of all new cancer cases in the world [1]. Smoking is recognized as a primary environmental risk factor of lung cancer, but only a fraction of smokers will develop lung cancer. Several studies observed that the incidence rate of lung cancer in non-smokers is increasing, especially for females in China. High incidence rates of lung cancer in Chinese female non-smokers appear to be related to other factors [2]. Therefore, exploring other related lung cancer risk factors in Chinese female non-smokers seems very meaningful.

The genesis and development of lung cancer is influenced by many risk factors, including genetic mutations and environmental factors and their interactions. Previous studies have confirmed many different genetic factors are involved in the development of lung cancer, including microRNA [3,4,5]. MicroRNAs are a type of single-stranded noncoding RNAs, the length of approximately 20 nucleotides, which are considered to regulate a large amount of gene expression mainly through binding to the 3’ untranslated region of their target mRNA [6]. The mature microRNA molecule will load together with several microRNA biosynthesis gene proteins, including at least one member of the *AGO* family, *Dicer*, *GEMIN3*, and *GEMIN4* into miRNA-induced silencing complex (miRISC) to play a critical role in silencing target mRNA. MicroRNAs have been confirmed to be involved in most of the human biological processes at the posttranscriptional level; deregulation of microRNA is considered to be involved in human cancer [7,8,9,10]. The abnormality of microRNA biosynthesis genes can affect the regulatory effect of global microRNAs to target mRNA, thereby influencing diseases; therefore, the abnormality of microRNA biosynthesis genes may play an important part in human cancer [11,12,13,14,15].

Recently, genetic association studies have identified that some genetic variants in microRNA biosynthesis genes may affect susceptibility to cancer risk such as gastric cancer, renal cell carcinoma, ovarian cancer, breast cancer and prostate cancer [16,17,18,19,20]. However the relationship between the SNPs in microRNA biosynthesis genes and the risk of lung cancer is still unclear. Herein, we evaluated the association between five SNPs in *GEMIN4 and AGO1*, the gene-environment interaction and lung cancer risk.

## 2. Materials and Methods

### 2.1. Study Subject

This study was approved by the Institutional Review Board of China Medical University. A total number of 868 participants consisting of 473 lung cancer cases and 395 cancer-free controls were included in the hospital-based case-control study. All participants were female non-smokers and genetically unrelated Chinese Han population. All participants signed informed consent. Patients were selected from the First Affiliated Hospital of China Medical University and the Liaoning Cancer Hospital. There was no restriction of age, clinical stage and histological type for the recruitment. All patients were newly diagnosed with histopathology-confirmed primary lung cancer that was previously untreated. During the same period, age matched (±5 years) cancer-free controls were recruited from medical examination centers in the same hospital.

### 2.2. Data Collection

A 10 mL venous blood sample was drawn from each subject and then stored at −20 °C for subsequent DNA isolation.

Clinical pathological information was obtained from clinical records. A face-to-face questionnaire interview was conducted among participants to collect demographics and environmental exposure information, including age, sex, smoking status, cooking oil fume exposure status and so on. In their lifetime, subjects who had smoked less than 100 cigarettes were defined as non-smokers, all others were smokers. Individuals who had been exposed to the secondhand smoke of one cigarette every day for at least one year were defined as passive smokers. For cooking oil fume exposure, participants were asked, “How often did the air in your kitchen become filled with oily ‘smoke’ during cooking?” There were four possible responses ranging from “never”, “seldom” and “sometimes” to “frequently”. Exposure to cooking oil fumes was defined as an indicator variable equal to 0 if participants reported seldom or never and equal to 1 if participants reported frequently or sometimes [21,22].

### 2.3. Genotyping Analysis

Genomic DNA was extracted from blood samples by the standard phenol-chloroform method. The genotyping method refers to our previous study [23].

### 2.4. Statistical Analysis

The Pearson chi-squared test was used to evaluate the Hardy-Weinberg equilibrium (HWE) in controls. The *t*-test and chi-squared test were separately performed to assess the distribution of continuous variables and categorical variables between two groups. The odds ratios (ORs) and their 95% confidence intervals (CIs) for assessing the relationship between risk factors and lung cancer risks were performed by logistic regression. The linkage disequilibrium (LD) and haplotype analyses were calculated by SHEsis online web-server [24]. The analysis of cumulative effects of unfavorable genotypes included those genotypes showing significant association with increased lung cancer risk in the main analysis. Crossover analysis was performed to assess gene-environment interaction. The evaluation of the additive interactions was based on Tomas Andersson’s study [25]. Multiplicative interactions were assessed by logistic regression model.

All statistical tests were two-sided, and nominal *p* < 0.05 was defined as statistically significant. The Bonferroni correction was used to adjust *p* value for multiple statistical tests. The SPSS 22.0 software (IBM, New York, NY, USA) was used for statistical analyses in the present study.

## 3. Results

### 3.1. Population Characteristics

The basic characteristics of participants are summarized in Table 1. The participants were composed of 473 lung cancer cases and 395 controls. All included individuals were Chinese female non-smokers, and no significant difference was found for age between two groups (*p* = 0.87).

### 3.2. Relationship between the Five Single Nucleotide Polymorphisms (SNPs) in GEMIN4 and AGO1 and Lung Cancer Risk

Table 2 lists the information of five SNPs. The observed genotype frequencies for each polymorphism among controls followed Hardy-Weinberg equilibrium (HWE) (*p* > 0.05).

First we assessed the association of the five SNPs and the lung cancer risk. Data is listed in Table 3. The results indicate that the distribution of rs7813, rs2291778, rs910924 and rs595961 genotypes exhibited statistically significant differences between two groups (*p* < 0.05); after Bonferroni correction, rs7813 and rs595961 were still associated with lung cancer risk.

Then, we conducted stratified analyses based on different histology types; the results on adenocarcinoma are shown in Table 4. From analysis between lung adenocarcinoma cases and controls, we found that the genotype and/or allele frequencies of the five SNPs were significantly different. After Bonferroni correction, significant association was maintained for rs7813, rs910924 and rs595961. Subjects carrying rs7813 CT (adjusted OR = 0.550, 95% CI = 0.401–0.755, *p* < 0.001) genotypes showed a decreased risk of lung adenocarcinoma compared to the subjects carrying homozygous TT genotype. The dominant genetic model (CT + CC) and additive model in rs7813 also showed a significant decrease in risk of lung adenocarcinoma with adjusted ORs of 0.562 (95% CI = 0.417–0.757, *p* < 0.001) and 0.689 (95% CI = 0.551–0.862, *p* = 0.001), respectively. Taking rs910924-CC genotype as a reference group, CT (adjusted OR = 0.540, 95% CI = 0.374–0.779, *p* = 0.001) genotype and dominant genetic model (CT + TT) (adjusted OR = 0.540, 95% CI = 0.379–0.769, *p* = 0.001) showed a significantly decreased risk of lung adenocarcinoma; T allele (adjusted OR = 0.583, 95% CI = 0.424–0.802, *p* = 0.001) was a protective allele in lung adenocarcinoma. The rs595961-A allele increased the lung adenocarcinoma risk in the additive model (adjusted OR = 1.502, 95% CI = 1.143–1.974, *p* = 0.003). Compared with the reference (rs595961-GG), AG and AG + AA were associated with a significantly increased risk of lung adenocarcinoma (adjusted OR = 1.580, 95% CI = 1.143–2.184, *p* = 0.006, adjusted OR = 1.609, 95% CI = 1.175–2.205, *p* = 0.003, respectively).

In squamous cell carcinoma, no significant differences were found between the distributions of genotypes in two groups. In SCLC, distribution of rs2291778 genotypes showed a remarkable result; however, due to the relatively small sample size, the results need to be further verified with a large sample population (Table S1).

### 3.3. The Linkage Disequilibrium (LD) and Haplotype Analyses of the SNPs in GEMIN4 s and Lung Cancer Risk

**Figure 1:**
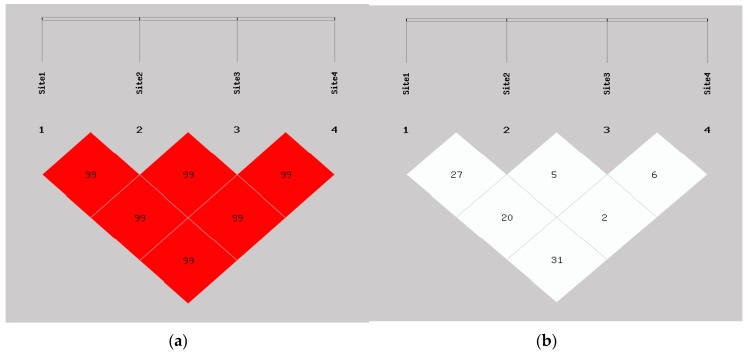
Linkage disequilibrium structure of four SNPs in *GEMIN4*. Site1 is rs7813. Site2 is 2740349. Site3 is rs2291778. Site4 is 910924. (**a**) D’ linkage map of four SNPs in *GEMIN4*; (**b**) R^2^ linkage map of four SNPs in *GEMIN4*.

We analyzed the association between different haplotypes and lung cancer risk. The LD plots are shown in Figure 1. Table 5 lists the frequencies of the haplotypes constructed with four SNPs: rs7813, rs2740349, rs2291778 and rs910924 in the *GEMIN4* gene. Five common haplotypes were also observed. Compared with the combination of all other haplotypes, C-A-G-T showed a protective effect in lung cancer and lung adenocarcinoma (OR = 0.688, 95% CI = 0.523–0.905, *p* = 0.007; OR = 0.583, 95% CI = 0.424–0.801, *p* = 0.001, respectively).

### 3.4. Cumulative Effects of the Unfavorable Genotypes in Lung Adenocarcinoma

As a result of the strong association between SNPs and risk of lung adenocarcinoma, we further assessed the combined effects of the high-risk genotypes on the lung adenocarcinoma risk (Table 6). The unfavorable genotypes were defined as following: rs7813 (TT), rs2740349 (AA), rs2291778 (GT + TT), rs910924 (CC), rs595961 (AG + AA). With the increasing number of unfavorable genotypes, a progressively increased gene-dose effect was found. The low-risk group’s subjects carrying zero/one unfavorable genotype were used as reference, whereas subjects carrying two/three and four/five unfavorable genotypes showed an increased risk of lung adenocarcinoma (adjusted OR = 1.798, 95% CI = 1.175–2.751, *p* = 0.007; adjusted OR = 3.206, 95% CI = 2.063–4.983, *p* < 0.001, respectively).

### 3.5. SNPs in GEMIN4 and AGO1 and Environmental Risk Factors (Cooking Oil Fume Exposure and Passive Smoking Exposure) as Well as Their Interaction on the Risk of Lung Cancer

Of the participants in this study, there were 224 cases and 244 controls with environmental exposure information. Individuals exposed to cooking oil fumes have a higher risk of lung cancer (OR = 2.132, 95% CI = 1.416–3.212, *p* < 0.001). Table 7 shows the interaction between environmental risk factors and these five SNPs on lung cancer risk. Compared with the reference group (rs595961-GG genotype carrier without environmental risk factors exposure), AG + AA genotype carriers exposed to cooking oil fumes or passive smoking have a significantly increased risk of lung cancer after Bonferroni correction (adjusted OR = 6.314, 95% CI = 2.752–14.485, *p* < 0.001, adjusted OR = 3.139, 95% CI = 1.678–5.871, *p* < 0.001, respectively).

The crossover analysis suggested the possibility of the existence of gene-environment interaction, so further analyses based on the additive scale (Table S2) and multiplicative scale were performed. The results suggest that there is no significant interaction on the additive scale. Logistic models were used to evaluate the interaction on a multiplicative scale; the results did not show any statistical significance.

## 4. Discussion

The relationship between SNPs of microRNA biosynthesis genes and the lung cancer risk has not been widely studied. To our knowledge, this is the first study to focus on the five SNPs of microRNA biosynthesis genes, cooking oil fumes and passive smoking exposure with risk of lung cancer. In order to control the influence of cigarette smoking on lung cancer, we selected this female non-smoker population as our study participants. It is noteworthy that the results of distribution of the SNPs, haplotype analysis and cumulative effects of the unfavorable genotypes all showed remarkable results in lung cancer. Meaningful results suggest that further functional studies need to be carried out to explore the underlying mechanisms of how the five SNPs affect lung cancer.

MicroRNA and some essential proteins, including GEMIN4 and AGO1, formed miRISC, through which the translation and stability of target mRNA were negatively regulated. miRISC play a role similar to oncogenes or tumor-suppressor genes involved in multiple tumor types by inhibiting the expression of target genes [26,27,28]. AGO family proteins contain three evolutionarily conserved domains, PAZ, MID and PIWI. The seed sequence of microRNA directly or indirectly anchored MID and PIWI domains in a deep pocket. Subsequently, GW182 family proteins directly act downstream of AGO proteins to affect miRNA-mediated repression. In miRISC, the AGO proteins serve as scaffolds to recruit GW182 to mRNA [29,30]. The *GEMIN4* gene has been mapped to chromosome 17p13 and encodes 1058 amino acids. The role of GEMIN4 protein in miRISC is not very clear. Aberrant microRNA biosynthesis genes have been found to be implicated in the genesis, development and survival of several types of cancer, indicating that a more general role may exist in microRNA biosynthesis genes in modifying the development of cancer [15,16,18,19,20,31,32]. While the underlying associations by which microRNA biosynthesis genes influences the risk of lung cancer remains unclear, our findings provide strong evidence regarding the association between SNPs in microRNA biosynthesis genes and lung cancer risk.

The nonsynonymous SNP rs7813 of the *GEMIN4* gene could induce Arg to Cys substitution at the 1033 amino acid position through the C to T transition. Interestingly, Liang et al. found that in the non-Hispanic Caucasian population, rs7813 and rs2740349 were at the top of 226 microRNA biosynthesis gene SNPs associated with ovarian cancer risk [18]. Our study found that the T allele of rs7813 has a negative effect on lung cancer risk. Our finding is identical with the other two studies on rs7813 and cancer risk [16,18]. However, the earliest study about rs7813 and cancer risk, that by Yang et al., evaluated the relationship between rs7813 and bladder cancer risk in the Caucasian population, though no significant association was found [33]. As rs910924 is located in the *GEMIN4* gene promoter region, we found that the CC genotype is an unfavorable genotype. Two previous studies about the relationship between rs910924 and cancer risk have not reached a statistically significant level [16,33]. In 2010, a study was carried out between 24 SNPs in 11 microRNA biosynthesis genes and lung cancer risk; the distribution of nine SNPs in *GEMIN4* and *AGO1* gene did not show any statistical difference between 100 cases and 100 controls [34].

According to previous studies, haplotypes are more meaningful than a single SNP for changes in gene function [35,36]. In our study, five common haplotypes were detected; after Bonferroni correction, one of them was still found to be associated with lung cancer risk. The analysis between cumulative effect of unfavorable genotypes and lung adenocarcinoma risk also showed a notable result. It is remarkable that our results revealed that the SNPs and haplotypes were more correlated with the lung adenocarcinoma risk than other types of lung cancer, suggesting that the function of SNPs of the *GEMIN4* gene may have cell specificity. This fact may signify that these SNPs provide genetic marker identification for different types of lung cancer. However, the sample size of lung adenocarcinoma and SCLC research was small, and the results need to be further verified in a larger sample population.

Lung cancer is a kind of malignant tumor which is affected by many factors, including genetic and environmental factors and their interactions. In this study, our results indicated that a higher risk of lung cancer was found in the cooking oil fume exposure group, but no gene-environment interaction was found. The results are consistent with our previous studies [37,38]. Relevant studies found that DNA damage can be induced by cooking oil fume exposure and influence the carcinogenesis and development of lung cancer [39,40]. Chinese cooking involves more high-temperature cooking and frying processes, so more cooking oil fumes will be produced. Cooking oil fumes contain large amounts of carcinogens, which is likely to play a part in the carcinogenesis and development of lung cancer. Further studies on the mechanisms behind and relationship of cooking oil fumes and lung cancer should be carried out.

This is the first study to show a significant association between microRNA biosynthesis genes polymorphism and lung cancer risk. There are some limitations to our study, however. First, the relatively small sample size may not have provided enough statistical power. Second, since this study was a hospital-based study, selection bias may exist. Third, other SNPs in microRNA biosynthesis genes may be involved in lung cancer risk. In addition, there are some other environmental risk factors involved in lung cancer that may not have been considered in the present study.

## 5. Conclusions

On the whole, the present study firstly reported the significant association between rs7813 and rs595961 and lung cancer risk. We also found that individuals exposed to cooking oil fumes showed a relatively high risk of lung cancer, although no interactions were found between environmental risk factor exposure and these SNPs.

## Figures and Tables

**Table 1:** Characteristics of lung cancer cases and cancer-free controls.

Variables	Cases (%)	Controls (%)	*p* Value
Females	473	395	
Mean age (years)	56.26 ± 11.71	56.13 ± 11.64	0.87
Histological			
Adenocarcinoma	321 (67.9%)		
Squamous cell carcinoma	65 (13.7%)		
SCLC	66 (14.0%)		
Others **^a^**	21 (4.4%)		

**^a^** Including adenosquamous carcinoma, mixed-cell and undifferentiated carcinoma.

**Table 2:** Single nucleotide polymorphisms in microRNA biogenesis genes.

*Chr* Location	Gene	SNP	Position	Major/Minor Allele
17p13	*GEMIN4*	rs7813	C1022R	T/C
17p13	*GEMIN4*	rs2740349	N918D	A/G
17p13	*GEMIN4*	rs2291778	Intron	G/T
17p13	*GEMIN4*	rs910924	Promoter	C/T
1p34.3	*AGO1(EIF2C1)*	rs595961	Intron	G/A

**Table 3:** Distribution of genotypes and ORs for lung cancer cases and cancer-free controls.

SNP	Genotype	Lung Cancer Cases (%) *N* = 473	Controls (%) *N* = 395	*p* of HWE	Adjusted OR ^a^	95% CI	*p*
rs7813	TT	242 (51.2)	153 (38.7)	0.320	Ref		
	CT	177 (37.4)	193 (48.9)		0.580	0.435, 0.773	**<0.001** *
	CC	54 (11.4)	49 (12.4)		0.694	0.448, 1.075	0.102
Dominant model	CT + CC	231 (48.8)	242 (61.3)		0.604	0.460, 0.792	**<0.001** *
Additive model	C allele				0.740	0.605, 0.904	**0.003** *
rs2740349	AA	375 (79.3)	298 (75.4)	0.123	Ref		
	AG	93 (19.7)	86 (21.8)		0.859	0.617, 1.195	0.367
	GG	5 (1.1)	11 (2.8)		0.361	0.124, 1.051	0.062
Dominant model	AG + GG	98 (20.7)	97 (24.6)		0.803	0.584, 1.106	0.179
Additive mode	G allele				0.772	0.579, 1.030	0.078
rs2291778	GG	225 (47.6)	214 (54.2)	0.513	Ref		
	GT	196 (41.4)	150 (38.0)		1.239	0.933, 1.645	0.139
	TT	52 (11.0)	31 (7.8)		1.596	0.983, 2.591	0.059
Dominant model	GT + TT	248 (52.4)	181 (45.8)		1.303	0.996, 1.704	0.053
Additive mode	T allele				1.265	1.027, 1.559	0.027 *
rs910924	CC	369 (78.0)	277 (70.1)	0.891	Ref		
	CT	96 (20.3)	108 (27.3)		0.667	0.487, 0.915	0.012 *
	TT	8 (1.7)	10 (2.5)		0.600	0.234, 1.541	0.289
Dominant model	CT + TT	104 (22.0)	118 (29.9)		0.662	0.487, 0.899	0.008 *
Additive mode	T allele				0.695	0.528, 0.913	0.009 *
rs595961	GG	293 (61.9)	285 (72.2)	0.748	Ref		
	AG	167 (35.3)	102 (25.8)		1.593	1.186, 2.141	**0.002** *
	AA	13 (2.7)	8 (2.0)		1.580	0.645, 3.871	0.317
Dominant model	AG + AA	180 (38.1)	110 (27.8)		1.592	1.194, 2.123	**0.002** *
Additive mode	A allele				1.460	1.135, 1.878	**0.003** *

^a^ Adjusted for age, ORs and 95% CIs were calculated by logistic regression. * *p* < 0.05. Bold values indicate significance after Bonferroni correction (*k* = 8).

**Table 4:** Distribution of genotypes and ORs for adenocarcinoma cases and cancer-free controls.

SNP	Genotype	Controls (%)	Adenocarcinoma (%)	Adjusted OR ^a^	95% CI	*p*
		*N* = 395	*N* = 321			
rs7813	TT	153 (38.7)	170 (53.0)	Ref		
	CT	193 (48.9)	118 (36.8)	0.550	0.401, 0.755	**<0.001** *
	CC	49 (12.4)	33 (10.3)	0.595	0.362, 0.976	0.040 *
Dominant model	CT + CC	242 (61.3)	151 (47.0)	0.562	0.417, 0.757	**<0.001** *
Additive model	C allele			0.689	0.551, 0.862	**0.001** *
rs2740349	AA	298 (75.4)	259 (80.7)	Ref		
	AG	86 (21.8)	59 (18.4)	0.788	0.543, 1.141	0.207
	GG	11 (2.8)	3 (0.9)	0.313	0.086, 1.134	0.077
Dominant model	AG + GG	97 (24.6)	62 (19.3)	0.734	0.512, 1.052	0.093
Additive model	G allele			0.711	0.513, 0.987	0.041 *
rs2291778	GG	214 (54.2)	150 (46.7)	Ref		
	GT	150 (38.0)	134 (41.7)	1.275	0.932, 1.745	0.128
	TT	31 (7.8)	37 (11.5)	1.719	1.017, 2.904	0.043 *
Dominant model	GT + TT	181 (45.8)	171 (53.3)	1.354	1.006, 1.821	0.045 *
Additive model	T allele			1.307	1.040, 1.642	0.021 *
rs910924	CC	277 (70.1)	261 (81.3)	Ref		
	CT	108 (27.3)	55 (17.1)	0.540	0.374, 0.779	**0.001** *
	TT	10 (2.5)	5 (1.6)	0.533	0.180, 1.582	0.257
Dominant model	CT + TT	118 (29.9)	60 (18.7)	0.540	0.379, 0.769	**0.001** *
Additive model	T allele			0.583	0.424, 0.802	**0.001** *
rs595961	GG	285 (72.2)	198 (61.7)	Ref		
	AG	102 (25.8)	112 (34.9)	1.580	1.143, 2.184	**0.006** *
	AA	8 (2.0)	11 (3.4)	1.982	0.783, 5.018	0.149
Dominant model	AG + AA	110 (27.8)	123 (38.3)	1.609	1.175, 2.205	**0.003** *
Additive model	A allele			1.502	1.143, 1.974	**0.003** *

^a^ Adjusted for age, ORs and 95% CIs were calculated by logistic regression; * *p* < 0.05. Bold values indicate significance after Bonferroni correction (*k* = 8).

**Table 5:** Haplotypes and the risk of lung cancer (rs7813–rs2740349–rs2291778–rs910924).

Haplotype ^a^	Controls (%)	Lung Cancer			Adenocarcinoma		
		N (%)	OR (95% CI)	*p*	N (%)	OR (95% CI)	*p*
TAGC	287 (36.3)	360 (38.1)	1.080 (0.889, 1.314)	0.438	250 (38.9)	1.118 (0.901, 1.386)	0.310
TATC	212 (26.8)	300 (31.7)	1.265 (1.027, 1.559)	0.027 *	208 (32.4)	1.307 (1.040, 1.642)	0.021 *
CAGT	128 (16.2)	111 (11.7)	0.688 (0.523, 0.905)	**0.007** *	65 (10.1)	0.583 (0.424, 0.801)	**0.001** *
CGGC	108 (13.7)	103 (10.9)	0.772 (0.579, 1.030)	0.078	65 (10.1)	0.711 (0.513, 0.986)	0.040 *
CAGC	55 (7)	71 (7.5)	1.087 (0.754, 1.566)	0.655	54 (8.4)	1.227 (0.830, 1.814)	0.303

^a^ Frequency of haplotypes < 3% were excluded from the final analysis; * *p* < 0.05. Bold values indicate significance after Bonferroni correction (*k* = 5).

**Table 6:** Cumulative effect of unfavorable genotypes and lung adenocarcinoma risk.

Number of Unfavorable Genotypes ^a^	Adenocarcinoma (%)	Controls (%)	Adjusted OR ^b^	95% CI	*p*
0/1	41 (12.8)	100 (25.3)	Ref		
2/3	137 (42.7)	186 (47.1)	1.798	1.175, 2.751	**0.007** *
4/5	143 (44.5)	109 (27.6)	3.206	2.063, 4.983	**<0.001** *

^a^ Unfavorable genotypes: rs7813 (TT), rs2740349 (AA), rs2291778 (GT + TT), rs910924(CC), rs595961(AG+AA); ^b^ Adjusted for age, ORs and 95% CIs were calculated by logistic regression; * *p* < 0.05; Bold values indicate significance after Bonferroni correction (*k* = 2).

**Table 7:** Interaction of five SNPs and environmental risk factors on lung cancer risk.

Cooking Oil Fume Exposure	Genotype	Cases (%)	Controls (%)	Adjusted OR ^a^	95% CI	*p* Value	Passive Smoking Exposure	Cases (%)	Controls (%)	Adjusted OR ^a^	95% CI	*p* Value
	rs7813											
−	CT + CC	68 (30.4)	118 (48.4)	Ref			−	46 (20.5)	62 (25.4)	Ref		
−	TT	74 (33.0)	74 (30.3)	1.836	1.073, 3.143	0.027 *	−	47 (21.0)	54 (22.1)	1.189	0.613, 2.305	0.609
+	CT + CC	46 (20.5)	36 (14.8)	2.266	1.182, 4.343	0.014 *	+	68 (30.4)	92 (37.7)	1.022	0.564, 1.851	0.940
+	TT	36 (16.1)	16 (6.6)	1.778	0.688, 4.598	0.235	+	63 (28.1)	36 (14.8)	2.578	1.266, 5.252	0.009 *
	rs2740349											
−	AG + GG	26 (11.6)	50 (20.5)	Ref			−	19 (8.5)	34 (13.9)	Ref		
−	AA	116 (51.8)	142 (58.2)	1.746	0.920, 3.313	0.088	−	74 (33.0)	82 (33.6)	1.586	0.739, 3.402	0.237
+	AG + GG	18 (8.0)	14 (5.7)	2.384	0.834, 6.808	0.105	+	25 (11.2)	30 (12.3)	1.493	0.596, 3.741	0.392
+	AA	64 (28.6)	38 (15.6)	1.316	0.472, 3.669	0.600	+	106 (47.3)	98 (40.2)	2.065	0.974, 4.379	0.059
	rs2291778											
−	GG	64 (28.6)	84 (34.4)	Ref			−	45 (20.1)	54 (22.1)	Ref		
−	GT + TT	78 (34.8)	108 (44.3)	0.992	0.583, 1.685	0.975	−	48 (21.4)	62 (25.4)	0.903	0.465, 1.752	0.762
+	GG	40 (17.9)	26 (10.7)	2.028	0.969, 4.245	0.061	+	59 (26.3)	56 (23.0)	1.263	0.655, 2.437	0.486
+	GT + TT	42 (18.8)	26 (10.7)	2.157	1.031, 4.512	0.041 *	+	72 (32.1)	72 (29.5)	1.325	0.700, 2.511	0.388
	rs910924											
−	CT + TT	32 (14.3)	56 (23.0)	Ref			−	20 (8.9)	30 (12.3)	Ref		
−	CC	110 (49.1)	136 (55.7)	1.437	0.788, 2.623	0.237	−	73 (32.6)	86 (35.2)	1.296	0.597, 2.814	0.512
+	CT + TT	19 (8.5)	18 (7.4)	1.860	0.721, 4.799	0.199	+	31 (13.8)	44 (18.0)	1.126	0.468, 2.707	0.792
+	CC	63 (28.1)	34 (13.9)	1.755	0.674, 4.572	0.249	+	100 (44.6)	84 (34.4)	1.863	0.864, 4.016	0.112
	Rs595961											
−	GG	93 (41.5)	144 (59.0)	Ref			−	56 (25.0)	84 (34.4)	Ref		
−	AG + AA	49 (21.9)	48 (19.7)	1.594	0.982, 2.587	0.059	−	37 (16.5)	32 (13.1)	0.978	0.955, 1.002	0.072
+	GG	50 (22.3)	44 (18.0)	1.726	1.060, 2.810	0.028 *	+	87 (38.8)	104 (42.6)	1.333	0.850, 2.091	0.210
+	AG + AA	32 (14.3)	8 (3.3)	6.314	2.752, 14.485	**<0.001** *	+	44(19.6)	24 (9.8)	3.139	1.678, 5.871	**<0.001** *

^a^ Adjusted for age, ORs and 95% CIs were calculated by logistic regression; * *p* < 0.05. Bold values indicate significance after Bonferroni correction (*k* = 6).

## Supplementary Materials

The following are available online at www.mdpi.com/1660-4601/13/10/939, Table S1. Distribution of genotypes and ORs for different types of lung cancer cases and cancer-free controls; Table S2. Interaction measures between SNPs and environmental risk factors on lung cancer risk.
