# Supplementary material for: Polymorphisms in GEMIN4 and AGO1 Genes Are Associated with the Risk of Lung Cancer: A Case-Control Study in Chinese Female Non-Smokers
Source: Int J Environ Res Public Health. 2016 Sep 23;13(10):939. doi: 10.3390/ijerph13100939 (PMC5086678; doi:10.3390/ijerph13100939)
Supplement: Supplementary file 1 [file ijerph-13-00939-s001.pdf]

Supplementary Materials: Polymorphisms in *GEMIN4* and *AGO1* Genes Are Associated with the Risk of Lung Cancer: A Case-Control Study in Chinese Female Non-Smokers

Xue Fang, Zhihua Yin, Xuelian Li, Lingzi Xia and Baosen Zhou

Table S1. Distribution of genotypes and ORs for different types of lung cancer cases and cancer-free controls.

SNP	Genotype	Controls (%)	Squamous Cell Carcinoma (%)			*p*	SCLC (%)			*p*
		(n = 395)	(n = 65)	Adjusted OR [a]	95% CI		(n = 66)	Adjusted OR [a]	95% CI	
rs7813	TT	153 (38.7)	29 (44.6)	Ref			30 (45.5)	Ref		
	CT	193 (48.9)	26 (40.0)	0.711	0.402, 1.257	0.241	26 (39.4)	0.684	0.388, 1.206	0.189
	CC	49 (12.4)	10 (15.4)	1.085	0.493, 2.388	0.839	10 (15.2)	1.037	0.470, 2.285	0.929
Dominant model	CT + CC	242 (61.3)	36 (55.4)	0.786	0.463, 1.335	0.372	36 (54.5)	0.750	0.443, 1.270	0.284
Additive model	C allele			0.942	0.639, 1.388	0.763		0.906	0.616, 1.335	0.619
rs2740349	AA	298 (75.4)	52 (80.0)	Ref			47 (71.2)	Ref		
	AG	86 (21.8)	12 (18.5)	0.799	0.408, 1.565	0.513	18 (27.3)	1.300	0.716, 2.358	0.389
	GG	11 (2.8)	1 (1.5)	0.512	0.065, 4.055	0.526	1 (1.5)	0.581	0.073, 4.605	0.607
Dominant model	AG + GG	97 (24.6)	13 (20.0)	0.769	0.402, 1.473	0.428	19 (28.8)	1.223	0.683, 2.188	0.498
Additive model	G allele			0.764	0.423, 1.380	0.373		1.112	0.662, 1.867	0.688
rs2291778	GG	214 (54.2)	40 (61.5)	Ref			26 (39.4)	Ref		
	GT	150 (38.0)	19 (29.2)	0.674	0.375, 1.209	0.186	33 (50.0)	1.829	1.047, 3.195	0.034 *
	TT	31 (7.8)	6 (9.2)	0.936	0.365, 2.414	0.895	7 (10.6)	2.109	0.828, 5.375	0.118
Dominant model	GT + TT	181 (45.8)	25 (38.5)	0.731	0.427, 1.253	0.255	40 (60.6)	1.876	1.097, 3.207	0.022 *
Additive model	T allele			0.843	0.547, 1.302	0.442		1.540	1.041, 2.278	0.031 *
rs910924	CC	277 (70.1)	41 (63.1)	Ref			48 (72.7)	Ref		
	CT	108 (27.3)	22 (33.8)	1.373	0.781, 2.413	0.271	17 (25.8)	0.895	0.492, 1.627	0.716
	TT	10 (2.5)	2 (3.1)	1.350	0.285, 6.380	0.705	1 (1.5)	0.551	0.069, 4.424	0.575
Dominant model	CT + TT	118 (29.9)	24 (36.9)	1.371	0.793, 2.372	0.259	18 (27.3)	0.871	0.486, 1.563	0.644

Table S1 Count.

Additive model	T allele			1.290	0.807, 2.064	0.288		0.865	0.513, 1.458	0.586
rs595961	GG	285 (72.2)	41 (63.1)	Ref			42 (63.6)	Ref		
	AG	102 (25.8)	23 (35.4)	1.573	0.900, 2.752	0.112	23 (34.8)	1.522	0.872, 2.656	0.140
	AA	8 (2.0)	1 (1.5)	0.873	0.106, 7.169	0.900	1 (1.5)	0.853	0.104, 6.999	0.883
Dominant model	AG + AA	110 (27.8)	24 (36.9)	1.522	0.878, 2.639	0.134	24 (36.4)	1.474	0.852, 2.550	0.165
Additive model	A allele			1.361	0.844, 2.196	0.206		1.326	0.823, 2.139	0.246

[a] Adjusted for age, ORs and 95% CIs were calculated by logistic regression; * $p < 0.05$

Table S2. Interaction measures between SNPs and environmental risk factors on lung cancer risk.

SNP	Cooking Oil Fume Exposure			Passive Smoking Exposure		
	Measurement	Estimate	95% CI	Measurement	Estimate	95% CI
rs7813	RERI	0.952	−1.288, 3.192	RERI	1.552	−0.556, 3.660
	AP	0.244	−0.256, 0.744	AP	0.635	0.086, 1.183
	S	1.488	0.568, 3.901	S	−13.434	-
rs2740349	RERI	0.195	−2.601, 2.992	RERI	−0.171	−1.689, 1.348
	AP	0.060	−0.791, 0.912	AP	−0.088	−0.859, 0.683
	S	1.096	0.286, 4.193	S	0.846	0.223, 3.211
rs2291778	RERI	−0.161	−2.375, 2.052	RERI	−0.007	−1.062, 1.048
	AP	−0.076	−1.140, 0.988	AP	−0.005	−0.781, 0.770
	S	0.875	0.146, 5.260	S	0.980	0.056, 17.308
rs910924	RERI	−1.070	−3.029, 0.890	RERI	0.456	0.285, 0.626
	AP	−0.897	−2.585, 0.791	AP	0.255	−0.167, 0.677
	S	0.153	0.003, 8.103	S	2.380	0.052, 110.005
rs595961	RERI	0.866	0.625, 1.107	RERI	2.550	−1.139, 6.239
	AP	0.078	−0.807, 6.962	AP	0.392	−0.011, 0.796
	S	0.453	0.352, 2.585	S	1.865	0.805, 4.324

RERI, the relative excess risk due to interaction; AP, the attributable proportion due to interaction; S, the synergy index.

© 2016 by the authors. Submitted for possible open access publication under the terms and conditions of the Creative Commons Attribution (CC-BY) license (http://creativecommons.org/licenses/by/4.0/).
